# Quantitative assessment of *BRAF* V600 mutant circulating cell-free tumor DNA as a tool for therapeutic monitoring in metastatic melanoma patients treated with BRAF/MEK inhibitors

**DOI:** 10.1186/s12967-016-0852-6

**Published:** 2016-04-19

**Authors:** Max Schreuer, Geert Meersseman, Sari Van Den Herrewegen, Yanina Jansen, Ines Chevolet, Ambre Bott, Sofie Wilgenhof, Teofila Seremet, Bart Jacobs, Ronald Buyl, Geert Maertens, Bart Neyns

**Affiliations:** Department of Medical Oncology, Universitair Ziekenhuis Brussel (UZ Brussel), Vrije Universiteit Brussel (VUB), Laarbeeklaan 101, 1090 Brussels, Belgium; Biocartis, Generaal De Wittelaan 11 B3, 2800 Mechelen, Belgium; Department of Dermatology, Universitair ziekenhuis Gent (UZ Gent), Universiteit Gent (UGent), De Pintelaan 185, 9000 Ghent, Belgium; Department of Biostatistics and Medical Informatics, Vrije Universiteit Brussel (VUB), Laarbeeklaan, 103, 1090 Brussels, Belgium

**Keywords:** Melanoma, cfDNA, ctDNA, *BRAF* V600, Biomarkers, Dabrafenib, Trametinib

## Abstract

**Background:**

*BRAF* V600 mutant circulating cell-free tumor DNA (*BRAF* V600mut ctDNA) could serve as a specific biomarker in patients with *BRAF* V600 mutant melanoma. We analyzed the value of *BRAF* V600mut ctDNA from plasma as a monitoring tool for advanced melanoma patients treated with BRAF/MEK inhibitors.

**Methods:**

Allele-specific quantitative PCR analysis for* BRAF *V600 E/E2/D/K/R/M mutations was performed on DNA extracted from plasma of patients with known *BRAF* V600 mutant melanoma who were treated with dabrafenib and trametinib.

**Results:**

245 plasma samples from 36 patients were analyzed. In 16 patients the first plasma sample was obtained before the first dosing of dabrafenib/trametinib. At baseline, *BRAF* V600mut ctDNA was detected in 75 % of patients (n = 12/16). *BRAF* V600mut ctDNA decreased rapidly upon initiation of targeted therapy (p < 0.001) and became undetectable in 60 % of patients (n = 7/12) after 6 weeks of treatment. During treatment, disease progression (PD) was diagnosed in 27 of 36 patients. An increase of the *BRAF* V600mut ctDNA copy number and fraction, identified PD with a sensitivity of 70 % (n = 19/27) and a specificity of 100 %. An increase in the *BRAF* V600mut ctDNA fraction was detected prior to clinical PD in 44 % of cases (n = 12/27) and simultaneously with PD in 26 % of patients (n = 7/27).

**Conclusions:**

Quantitative analysis of *BRAF* V600mut ctDNA in plasma has unique features as a monitoring tool during treatment with BRAF/MEK inhibitors. Its potential as an early predictor of acquired resistance deserves further evaluation.

## Background

The detection of mutations in circulating cell-free tumor DNA (ctDNA) is under investigation as a specific biomarker for the diagnosis and monitoring of patients with different cancer types [1–4]. Mutations in the *BRAF* gene at position V600 are detected in 40–50 % of cutaneous melanomas, and represent the most common oncogenic driver mutation in this disease [5]. Therefore, quantitative measurement of *BRAF* V600 mutant ctDNA in cell-free DNA (cfDNA) extracted from plasma could serve as a specific biomarker in this patient population [6, 7].

Treatment with a combination of a BRAF- and MEK inhibitor results in a high tumor response rate (64–69 %) and improves the survival of patients with *BRAF* V600 mutant melanoma [8–10]. Immune-checkpoint inhibition of either the CTLA-4 or PD-1 receptor can also improve the survival of patients with advanced melanoma, irrespective of the *BRAF* V600 mutation status [11–13]. Optimal sequencing of available treatment options for patients with *BRAF* V600 mutant melanoma has not been defined. Retrospective series have raised concern that ipilimumab may have lesser activity when applied after the emergence of resistance to BRAF-inhibitors [14, 15]. Alternatively, ipilimumab fails to improve the survival of patients with a life-expectancy of less than 3–4 months from initiating therapy, and durable complete responses have been reported on ipilimumab in patients who developed prior resistance to treatment with a BRAF-inhibitor [16]. Of concern is the high incidence of progression within the central nervous system (CNS) at first progression on BRAF-inhibitors, as metastases to the CNS often imply a poor prognosis and necessitate the use of corticotherapy, implying an unfavorable condition for initiating immunotherapy [17, 18]. As conventional clinical tools for assessment of early tumor progression lack sensitivity, many patients will be symptomatic at the time of progression or will experience rapid progression and deterioration in the few weeks that follow the diagnosis of progression [17, 19–21]. Therefore, more sensitive tools for monitoring of response and resistance to BRAF/MEK targeted therapy is of interest in order to optimize treatment of advanced *BRAF* V600 mutant melanoma. Furthermore, changes in the *BRAF* V600mut ctDNA concentration might be helpful for the interpretation of imaging results during immunotherapy where atypical tumor responses are more frequent [22, 23].

In this translational research study we analyze the value of longitudinal quantitative measurement of *BRAF* V600mut ctDNA from plasma as a therapeutic monitoring tool for patients with advanced *BRAF* V600 mutant melanoma treated with the BRAF/MEK inhibitors dabrafenib and trametinib.

## Methods

This was an exploratory translational study with a primary objective of investigating longitudinal quantitative measurement of *BRAF* V600mut ctDNA in patients treated with a combination of a BRAF and a MEK inhibitor using the Idylla™ ctBRAF Mutation prototype assay on the Idylla™ system (Biocartis). The study was conducted at a single university hospital (UZ Brussel, academic study sponsor) in collaboration with Biocartis (Mechelen, Belgium).

Patients were eligible for plasma *BRAF *V600mut ctDNA monitoring when a *BRAF* V600 mutation had been detected in tumor tissue and were either treated or initiated treatment with dabrafenib and trametinib. Blood samples were prospectively collected after obtaining informed consent with an Ethical Committee approved document. Response evaluation to targeted therapy was performed every 2 months with standard imaging techniques [including 18-fluorodeoxyglucose positron emission tomography/computed tomography (FDG PET–CT), computed tomography (CT) of thorax and abdomen, magnetic resonance imaging (MRI) of the brain]. Plasma samples were collected together with routine blood collections (every 2 weeks during the first month of therapy; every month after the first month of therapy), until progressive disease (PD) was detected according to RECIST v1.1 [24].

Blood samples were collected in 10 mL EDTA tubes and immediately centrifuged at a relative centrifugal force of 1410.63×*g*, during 15 min at room temperature. Plasma was separated and stored in 1-mL aliquots at −80 °C. Silica-based extraction of DNA and subsequent allele-specific quantitative PCR (qPCR) to detect the *BRAF* wild-type gene and the G1798>A and T1799>A changes in the *BRAF* gene were performed with Idylla™ (Biocartis) on 1 mL of the stored plasma. The G1798>A change is present in patients with V600 K, V600R, and V600 M mutations, whereas the T1799>A change is present in patients with V600E, V600 K, V600E2, and V600D mutations. A linear correlation between Cq values reported by prototype Idylla assays and digital droplet PCR was previously established, allowing precise and sensitive quantification of mutant ctDNA fragments in plasma down to 3 mutant copies per PCR reaction with an analytical sensitivity of 0.01 %. The investigators that performed the DNA extraction and the subsequent qPCR were blinded for the clinical patient information. Quantitative PCR results were expressed as median and 25th/75th percentiles of the *BRAF* V600mut ctDNA copy number per milliliter, and the *BRAF* V600mut ctDNA fraction to the total amount of cfDNA. The evolution of the *BRAF* V600mut ctDNA copy number and fraction, were analyzed in relation to the clinical diagnosis of PD. Progression-free survival (PFS) was measured from the time of first administration of targeted therapy, to the time of PD or death, and was analyzed using the Kaplan–Meier method (SPSS software, IBM). For comparison of PFS between different groups, the log-rank test was used. We applied the Wilcoxon signed-rank test to compare changes in the concentrations during treatment. Fisher’s exact test was used to compare categorical variables. A 2-tailed p value ≤0.05 was considered to be statistically significant. Statistical analysis was performed with SPSS statistics 22.

To assess the early evolution of the *BRAF* V600mut ctDNA concentration during treatment with dabrafenib and trametinib, five samples were collected during the first 24 h of treatment in two additional patients treated “in hospital”. In these two patients, all plasma samples were analyzed in duplicate. The first sample of 1 mL was analyzed with the Idylla™ system using a fully automated cartridge that contains all reagents for the above mentioned extraction of DNA followed by qPCR detection and reporting of the results. In the second sample of 1 mL, DNA was extracted using the QIAamp Circulating Nucleic Acid Kit (Qiagen), followed by droplet digital PCR (ddPCR, Bio-rad) for detection of *BRAF* V600Emut ctDNA. Each ddPCR analysis was performed in triplicate. Five μL of eluate was added to each ddPCR reaction containing 11.5 μL ddPCR Supermix for probes (Bio-Rad) and 3 μL of *BRAF* V600E and wild-type assay (dHsaCP2000027, dHsaCP2000028, Bio-Rad), adjusted to a total volume of 23 μL. The reaction was then compartmentalized into ~15,000 droplets per sample in a QX-100 droplet generator (Bio-Rad) according to the manufacturer’s instructions. Emulsified PCR reactions were run in 96-well format on a Bio-rad CFX96 Touch Real-Time PCR Detection System according to the manufacturer’s instructions. Plates were subsequently read on a Bio-Rad QX-100 droplet reader using QuantaSoft software (Bio-Rad). No-template controls were included in each run and the *BRAF* V600E copies per sample were quantified using the Quantasoft software.

## Results

### Patients and procurement of plasma samples

From February 2014 to October 2015, 245 plasma samples from 36 patients with advanced melanoma were collected. Clinical baseline patient characteristics are summarized in Table 1. In 16 patients the first plasma sample was obtained before the first dosing of dabrafenib (150 mg BID) and trametinib (2 mg QD). In 20 patients the first sample was obtained after initiation of targeted therapy.

**Table 1:** Patient baseline characteristics (n = 36)

Variable	Overall
Age	52 (12)
Sex	
Male	12 (33 %)
Female	24 (67 %)
Stage	
IVa	4 (11 %)
IVb	2 (6 %)
IVc	30 (83 %)
Primary site	
Cutaneous	28 (78 %)
Acral	4 (11 %)
Mucosal	0 (0 %)
Unknown	4 (11 %)
Treatment	
Dabrafenib + trametinib	34 (94 %)
Dabrafenib	2 (6 %)
Vemurafenib	0 (0 %)
Re-challenge dabrafenib + trametinib^a^	8 (22 %)
First plasma sample	
Before initiation of targeted therapy	16 (44 %)
After initiation of targeted therapy	20 (56 %)

Data are mean (SD) or n (%).

Baseline characteristics at the moment of collection of the first plasma sample

^a^Eight patients were treated with dabrafenib and trametinib after documentation of disease progression at least 12 weeks following the last day of dosing of a BRAF-inhibitor containing treatment regimen (ClinicalTrials.gov Identifier:NCT02296996)

### Early changes in *BRAF* V600 mutant ctDNA after treatment initiation with dabrafenib/trametinib

**Fig. 1:**
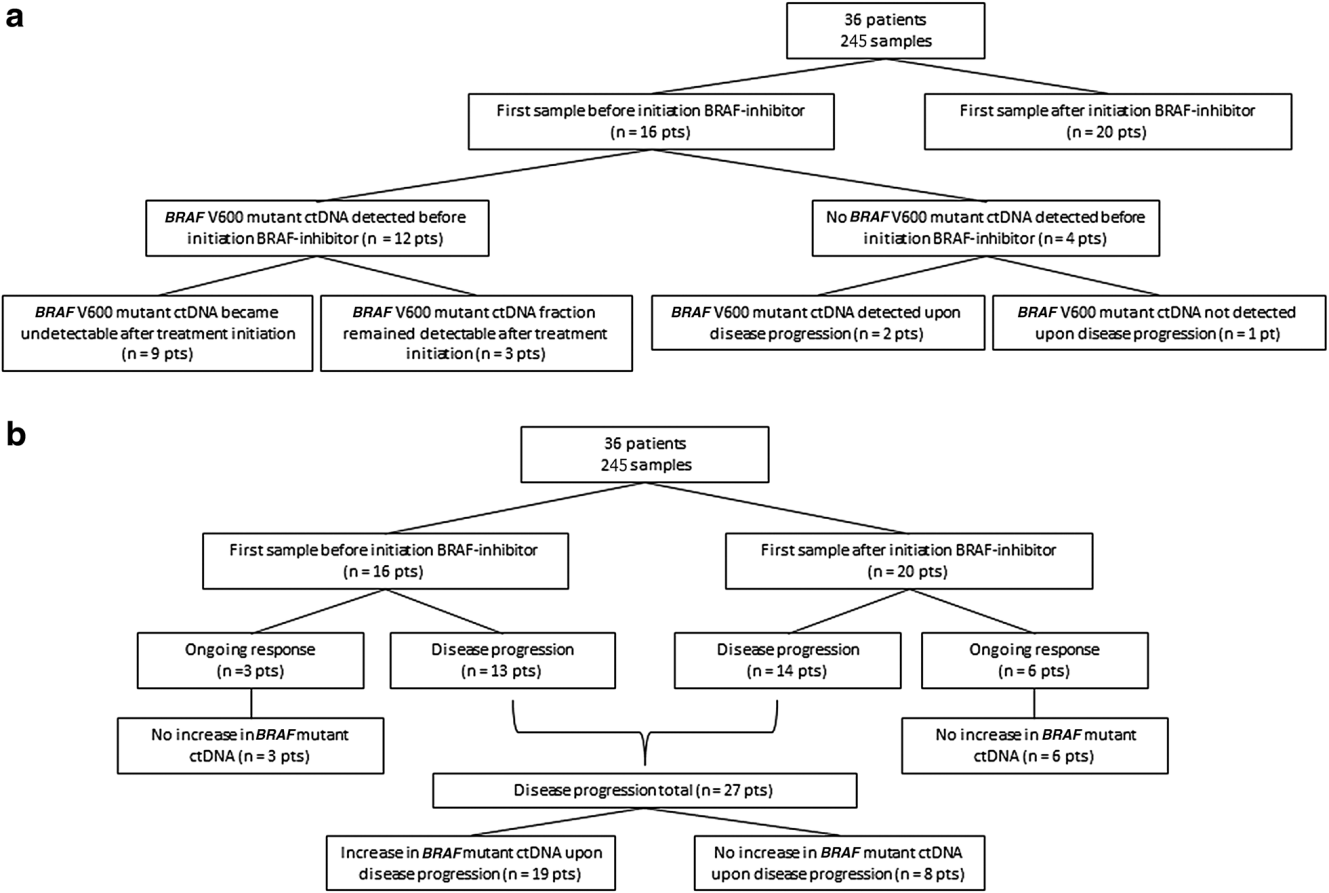
**a** Flow diagram showing subgroups of patients according to the detection of *BRAF* V600mut ctDNA after treatment initiation. **b** Flow diagram showing subgroups of patients according to the evolution of *BRAF* V600mut ctDNA in relation to disease progression. *Pts* patients

In 75 % of patients (n = 12/16) *BRAF* V600mut ctDNA was detected before initiation of dabrafenib and trametinib (median fraction 16.5 %; Q1–Q3 9.8–25 %—median copy number 571 copies/mL; Q1–Q3 79–1610 copies/mL) (Fig. 1a). In 25 % of patients (n = 4/16) no *BRAF* V600mut ctDNA was detected before initiation of dabrafenib and trametinib.

**Fig. 2:**
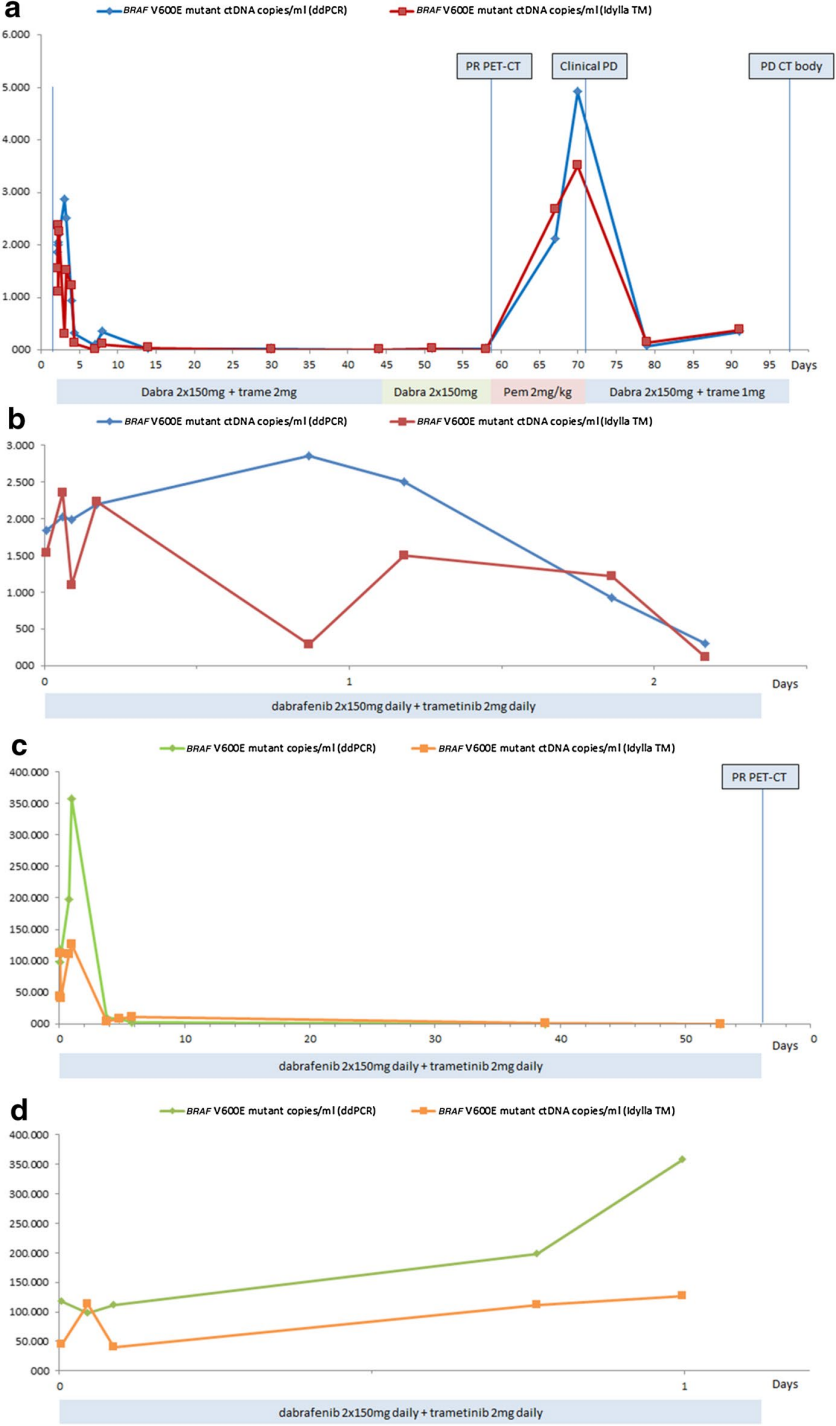
Serial measurement of *BRAF* V600mut ctDNA in plasma of 2 patients (**a**, **b** = patient 1; **c**, **d** = patient 2) with advanced melanoma during targeted therapy. The* y-axis* represents the *BRAF* V600mut ctDNA copy number per millilitre. Results obtained with the Idylla™ system (Biocartis) and digital PCR (Bio-rad) are shown. ** a** Treatment with dabrafenib and trametinib (dabra 2 × 150 mg + trame 2 mg) was initiated after obtaining the baseline plasma sample. Within 1 week the *BRAF* V600mut ctDNA copy number dropped significantly. After switching targeted therapy to pembrolizumab (Pem 2 mg/kg) due to side effects on the moment of best response (PR PET–CT), an increase of the BRAF v600mut ctDNA copy number was detected within 9 days. After reintroducing dabrafenib and trametinib, due to rapid clinical disease progression (Clinical PD), the *BRAF* V600mut ctDNA copy number dropped again. **b** The evolution of the *BRA* *F* V600mut ctDNA copy number in patient 1 during the first 2 days of treatment. **c** Treatment with dabrafenib and trametinib (dabra 2 × 150 mg + trame 2 mg) was initiated after obtaining the baseline plasma sample. Within 1 week the *BRAF* V600mut ctDNA copy number dropped significantly. **d** The evolution of the *BRAF* V600mut ctDNA copy number in patient 2 during the first day of treatment

To assess changes in the *BRAF* V600mut ctDNA concentration during the first hours after treatment initiation, five samples were collected in the first 24 h of treatment in 2 patients treated ‘‘in hospital’’ (Fig. 2). In both patients the *BRAF* V600mut ctDNA copy number decreased significantly within 5 days after treatment initiation. In one patient only a transient increase in the *BRAF* V600mut ctDNA copy number was observed within the first 24 h on therapy (Fig. 2b, d).

**Fig. 3:**
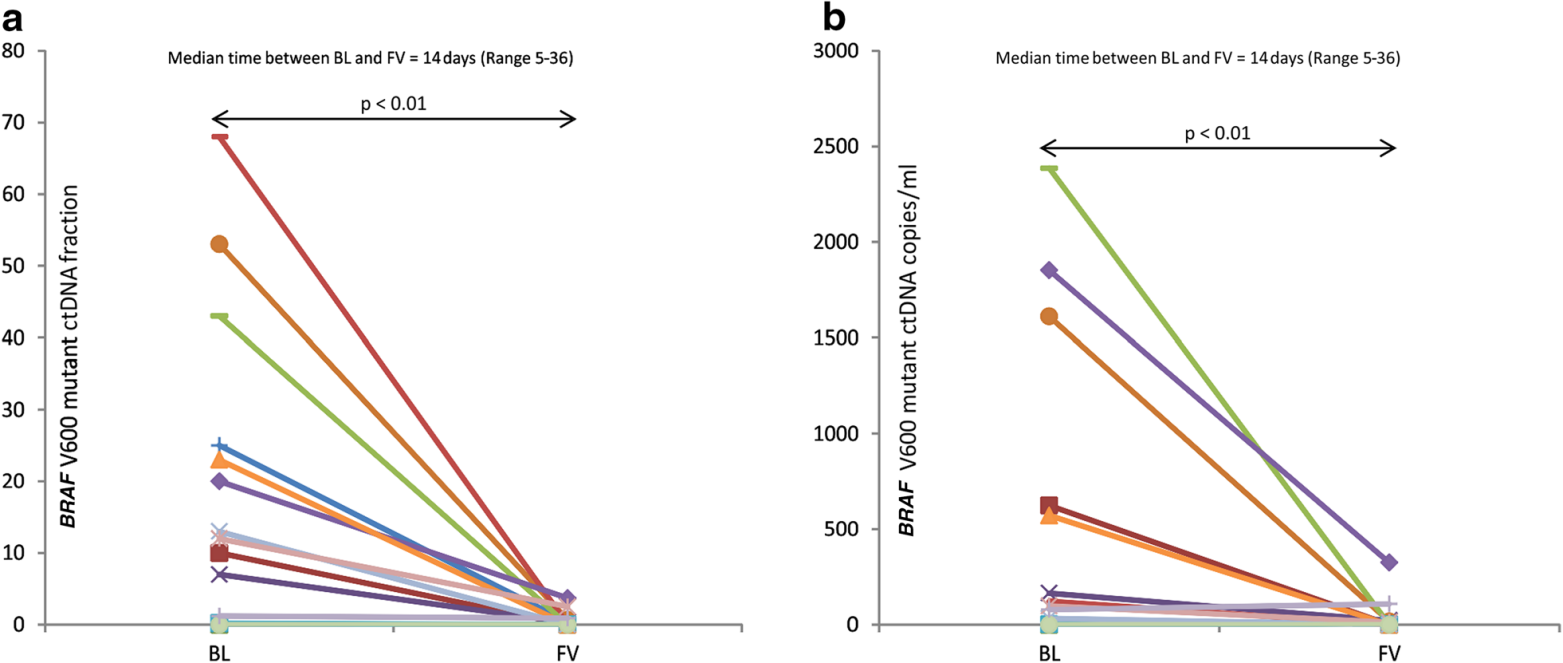
**a** Individual evolutions in the *BRAF* V600mut ctDNA fraction from initiation of treatment with dabrafenib (150 mg BID) and trametinib (2 mg QD), at baseline (BL) to the first visit (FV). **b** Individual evolutions in the *BRAF* V600mut ctDNA copy number from initiation of treatment with dabrafenib (150 mg BID) and trametinib (2 mg QD) at baseline (BL) to the first visit (FV)

**Fig. 4:**
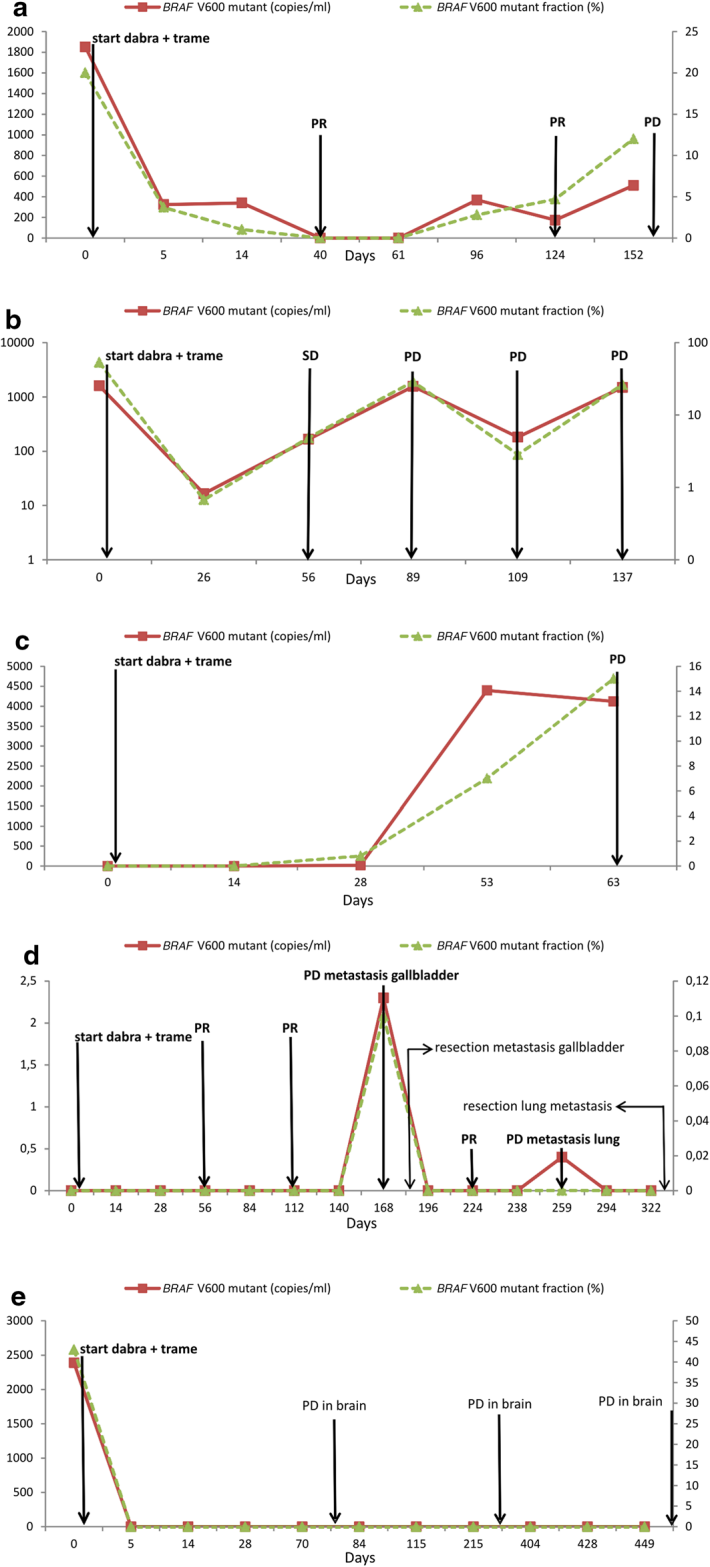
Serial measurement of *BRAF* V600mut ctDNA from plasma in 5 patients (**a**–**e**) with advanced melanoma during targeted therapy. Treatment with dabrafenib (dabra, 150 mg BID) and trametinib (trame, 2 mg QD) was initiated after obtaining the baseline plasma sample. The left* y-axis* represents the *BRAF* V600mut ctDNA copy number per milliliter (*solid line*), the right y-axis represents the *BRAF* V600mut ctDNA fraction to the total amount of cfDNA (*dashed line*). SD, PR and PD respectively denote stable disease, partial response and progressive disease according to RECIST v1.1. **a** The *BRAF* V600mut ctDNA copy number and fraction dropped after treatment initiation. After 40 days, no *BRAF* V600mut ctDNA could be detected anymore, and CT body showed a PR. The *BRAF* V600mut ctDNA fraction reappeared after 96 days and increased on day 124, although an ongoing PR was reported on PET–CT. PET–CT showed PD, 50 days after the reappearance of *BRAF* V600mut ctDNA in plasma. **b** *BRAF* V600mut ctDNA remained detectable from treatment initiation until PD was detected after 89 days. **c** At baseline, no *BRAF* V600mut ctDNA was detected in plasma. *BRAF* V600mut ctDNA appeared after 28 days, 35 days prior to the detection of PD on CT body. **d** At baseline, no *BRAF* V600mut ctDNA was detected in plasma. After 168 days, PD was detected in a gallbladder metastasis, and *BRAF* V600mut ctDNA was detected concomitantly. The only other lesion, a lung metastasis, had remained strictly stable. After resection of the gallbladder metastasis, the *BRAF* V600mut ctDNA fraction could not be detected until PD occurred in the remaining lung metastasis. **e** Five days after treatment initiation, *BRAF* V600mut ctDNA could not be detected anymore. Brain MRI showed a new millimetric brain lesion on the first response evaluation, while PET–CT showed clear regression of all liver and lung metastases. Subsequent MRI’s showed ongoing slow PD in the brain, while liver and lung lesions showed ongoing PR. No *BRAF *V600mut ctDNA was detected during PD in this patient

**Fig. 5:**
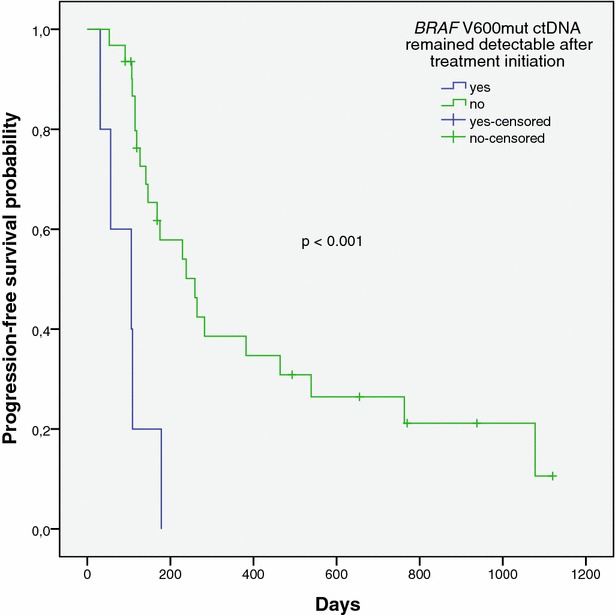
Kaplan–Meier plot representing the progression-free survival probability for patients with advanced melanoma in whom *BRAF* V600mut ctDNA became undetectable after initiation of a BRAF inhibitor containing treatment regimen (*solid line*), and for patients in whom *BRAF* V600mut ctDNA remained detectable during follow-up (*dashed line*)

During treatment, the *BRAF* V600mut ctDNA fraction and copy number decreased significantly compared to baseline in all 12 patients (p < 0.01, Fig. 3), and became undetectable (n = 7) or <1 % (n = 5) after a median of 13 days (range 6–40 days). At the time when no ctDNA could be measured any more, none of the patients had obtained a complete radiological remission (e.g. Fig.  4a, e). In 3 patients, *BRAF* V600mut ctDNA remained detectable from the initiation of targeted therapy until PD was diagnosed (e.g. Fig. 4b). Median PFS was not significantly longer for patients in whom *BRAF* V600mut ctDNA became undetectable 1 month after the initiation of targeted therapy, as compared to patients in whom ctDNA remained detectable during the first month of therapy (p = 0.56). Progression free survival was significantly shorter for patients in whom *BRAF* V600mut ctDNA remained detectable throughout the treatment with targeted therapy compared to patients in whom *BRAF* V600mut ctDNA became undetectable (p < 0.001) (Fig. 5).

### Correlation between *BRAF* V600 mutant ctDNA levels and disease progression

**Fig. 6:**
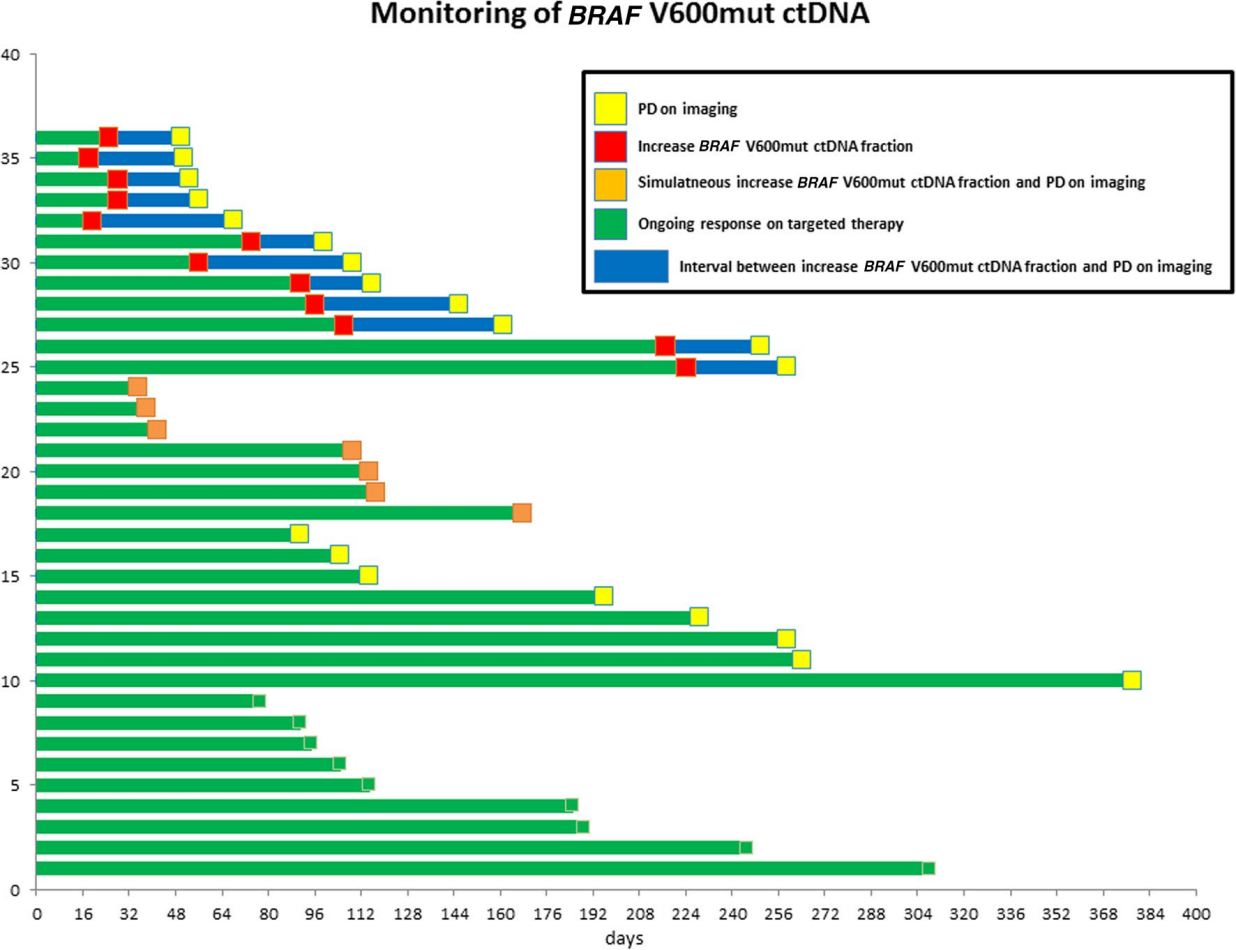
Swimmer plot showing the interval between increase of the *BRAF* V600mut ctDNA fraction and progressive disease according to RECIST v1.1 on imaging (PD), for all 36 patients patients from start of ctDNA monitoring. In 12 patients, an increase in the *BRAF* V600mut ctDNA fraction was detected prior to PD. In 7 patients, an increase of the *BRAF* V600mut ctDNA fraction was detected simultaneously with PD. In 8 patients, no increase in the *BRAF* V600mut ctDNA fraction was detected prior to or simultaneously with PD. None of the patients with an ongoing response showed an increase of the *BRAF* V600mut ctDNA fraction

During the course of plasma *BRAF* V600mut ctDNA monitoring, clinical PD was diagnosed in 27 of 36 patients after a median of 111 days [95 % CI 98–124] (Figs. 1b, 6). An increase of the *BRAF* V600mut ctDNA copy number and fraction was diagnosed in 19 of 27 (70 %) patients with PD and in none of the patients with an ongoing response, resulting in a sensitivity of 70 % and a specificity of 100 % (Kappa 0.54 [0.26–0.82]).

In 30 % (n = 8/27) of patients, no *BRAF* V600mut ctDNA was detected at the time of clinical PD. Of these eight patients, three showed PD in the brain (2 asymptomatic, e.g. Fig. 4e) and five showed slow PD in a single subcutaneous, bone, muscle or lung lesion.

An increase in the *BRAF* V600mut ctDNA fraction was detected prior to the clinical diagnosis of PD in 12 out of 27 (44 %) patients and simultaneously with PD in 7 out of 27 (26 %) patients. The first increase in the *BRAF* V600mut ctDNA fraction, after the *BRAF* V600mut ctDNA fraction became undetectable or was decreasing, was detected after a median of 74 days [95 % CI 19–130] from the collection of the first plasma sample. The median interval between the first increase in the *BRAF* V600mut ctDNA fraction and PD was 25 days [95 % CI 1–48 days]. A first increase in the *BRAF* V600mut ctDNA fraction or copy number predicted PD within the following month in 63 % of patients (n = 12/19; p < 0.001) and within the following 2 months in 100 % of patients (n = 19/19; p < 0.001).

When *BRAF* V600mut ctDNA could not be detected in plasma (n = 173 samples), PD did not occur in the following month in 86 %, and not in the following 2 months in 76 % of cases. Out of the four patients with no detectable *BRAF* V600 ctDNA at baseline, three patients showed PD during follow-up, and in two of these patients *BRAF* V600mut ctDNA was detected simultaneously with or before prior to PD on imaging. (Fig. 4c, d).

## Discussion

In this study, *BRAF* V600mut ctDNA was detected at baseline in 75 % of stage IV melanoma patients with a known *BRAF* V600 mutation. This is in line with two other studies using PCR based methods, where *BRAF* V600mut ctDNA was detected in plasma of 73–84 % of *BRAF* V600 mutant melanoma patients [6, 25]. These results are also in line with an overall detection rate of 77 % in 4 clinical studies with 746 stage IV *BRAF* V600 mutant melanoma patients using BEAMing (beads, emulsions, amplification, and magnetics analysis) after cfDNA extraction from plasma [26–30]. Moreover, one study showed a 100 % agreement between digital PCR, qPCR and BEAMing, suggesting that the suboptimal detection rate of *BRAF* V600 mutant DNA in plasma in advanced melanoma is the result of a variable amount of *BRAF* V600mut ctDNA in advanced melanoma patients, rather than an variability in the sensitivity of different test-platforms [25]. We observed that after initiating BRAF/MEK inhibitor treatment, *BRAF* V600mut ctDNA can drop below the detection limit, notwithstanding the absence of radiological complete remission. This phenomenon of rapid decrease in the concentration of *BRAF* V600mut ctDNA has been reported before and has been attributed to the destruction of tumor cells and a subsequent rapid clearance of ctDNA [6, 31–33]. Our combined observations of (1) decrease of the *BRAF* V600mut ctDNA concentration within days after treatment initiation, (2) of early increase during disease progression and (3) of early increase after discontinuation of targeted therapy, are suggestive of a correlation between ctDNA levels and the proliferation of melanoma cells. Therefore *BRAF* V600mut ctDNA seems to reflect the *BRAF* V600mut-dependent *proliferative* tumor burden, and not tumor mass as evidenced by CT-imaging. From this perspective, ctDNA could be attributed to increased cell death during melanoma cell proliferation or might be actively secreted or passively released by living cells, rather than by apoptotic or necrotic cells [34]. This latter hypothesis is supported by several preclinical arguments. Spontaneous active DNA release or apoptosis have been put forward as the main source of ctDNA, because plasma DNA often presents a ladder pattern, typical for active cleaving, when subjected to electrophoresis [32, 35]. Several arguments support the hypothesis that DNA is primarily released by living cancer cells rather than by apoptotic cancer cells: (1) DNA concentration increases in normal lymphocyte cultures following stimulation with phytohemagglutinin, lipopolysaccharide or antigen; (2) in a leukemic cell-line malignant cells released newly synthesized DNA; (3) cancer cell DNA concentration in cell culture supernatant increases with cell proliferation when few apoptotic or necrotic cells are present; and (4) circulating exosomal *BRAF* V600E mutant DNA was isolated from SK-MEL-28 melanoma-bearing mice [32, 33, 36, 37].

In our study population, PFS was significantly better for the patients in whom *BRAF* V600mut ctDNA became undetectable after the initiation of targeted therapy. When the *BRAF* V600mut ctDNA fraction increased during treatment, clinical PD always followed within 2 months. Moreover, in 44 % of cases an increase in the mutant fraction preceded PD. We recently reported that reappearance of *BRAF* V600mut ctDNA in plasma cfDNA can precede changes on PET–CT in patients under BRAF/MEK inhibitor treatment [34]. In this study an increase in the *BRAF* V600mut ctDNA fraction preceded PD with a median interval of 25 days. However, plasma samples were collected on a monthly basis, whereas imaging was performed every 2 months. In a study on ctDNA monitoring in metastatic breast cancer, increasing levels of ctDNA appeared on average 5 months before the establishment of PD by means of imaging [3].

An increase in the *BRAF* V600mut ctDNA fraction during BRAF/MEK targeted therapy could potentially serve as a trigger for early evaluation with imaging techniques, and allow for a timely switch to immunotherapy or other appropriate therapeutic interventions (e.g. stereotactic radiotherapy to brain metastases). Early detection of progression is important in metastatic melanoma, because of the aggressive course of the disease as exemplified by the observation that the central nervous system is a frequent site (30 %) of first progression under BRAF inhibitor therapy [17]. Moreover, potential second line therapeutic options such as ipilimumab and, to a lesser extent anti-PD-1 therapies, are associated with a latency of their anti-tumor effect. Therefore, the possibility of offering immunotherapy may be compromised if PD is only detected on imaging or when clinical symptoms are present [19, 20].

## Conclusions

Our results indicate that quantitative analysis of *BRAF* V600mut ctDNA in plasma holds promise as a monitoring tool in *BRAF* V600 mutant melanoma during treatment with BRAF/MEK targeted therapy. Prospective trials are needed to assess if an add-on strategy or switch to immunotherapy upon early detection of therapy resistance through ctDNA evolution, adds value to the current treatment strategies. Finally we provide clinical arguments suggesting that an important fraction of *BRAF* V600mut ctDNA correlates with the *BRAF* V600mut-dependent *proliferative* tumor burden.

## Abbreviations

*BRAF* V600mut ctDNA: *BRAF* V600 mutant circulating cell-free tumor DNA
ctDNA: circulating cell-free tumor DNA
cfDNA: circulating cell-free DNA
CNS: central nervous system
FDG PET–CT: fluorodeoxyglucose positron emission tomography/computed tomography
CT: computed tomography
MRI: magnetic resonance imaging
PD: progressive disease
qPCR: allele-specific quantitative PCR
PFS: progression-free survival
BID: bis in die (twice daily)
QD: quaque die (once daily)
BEAMing: beads, emulsions, amplification, and magnetics analysis
CR: complete remission
